# Polymer Films of 2-(Azulen-1-yldiazenyl)-5-(thiophen-2-yl)-1,3,4-thiadiazole: Surface Characterization and Electrochemical Sensing of Heavy Metals †

**DOI:** 10.3390/molecules30193959

**Published:** 2025-10-02

**Authors:** Cornelia Musina (Borsaru), Mihaela Cristea, Raluca Gavrilă, Oana Brincoveanu, Florin Constantin Comănescu, Veronica Anăstăsoaie, Gabriela Stanciu, Eleonora-Mihaela Ungureanu

**Affiliations:** 1 Faculty of Chemical Engineering and Biotechnologies, National University of Science and Technology Politehnica Bucharest, Gh. Polizu 1-7, Sector 1, 011061 Bucharest, Romania; borsaru_cornelia@yahoo.com; 2 “C. D. Nenitzescu” Institute of Organic and Supramolecular Chemistry, Romanian Academy, 71141 Bucharest, Romania; mihcris2012@yahoo.ro; 3 National Institute for Research and Development in Microtechnologies (IMT-Bucharest), 077190 Bucharest, Romania; raluca.gavrila@imt.ro (R.G.); oana.brincoveanu@imt.ro (O.B.); florin.comanescu@imt.ro (F.C.C.); 4 Department of Chemistry and Chemical Engineering, “Ovidius” University Constanta, 900527 Constanta, Romania; gstanciu@univ-ovidius.ro; 5 Doctoral School of Chemical Engineering and Biotechnologies, Faculty of Chemical Engineering and Bio-Technologies, National University of Science and Technology Politehnica Bucharest, Gh. Polizu 1-7, Sector 1, 011061 Bucharest, Romania

**Keywords:** 2-(azulen-1-yldiazenyl)-5-(thiophen-2-yl)-1,3,4-thiadiazole, electrochemical methods, sensitive modified electrodes, electropolymerization, heavy metal detection

## Abstract

This work introduces 2-(azulen-1-yldiazenyl)-5-(thiophen-2-yl)-1,3,4-thiadiazole (**L**) as a functional monomer capable of forming stable, redox-active films with high affinity for lead in aqueous solutions. **L** was synthesized and characterized using physical chemical methods and electrochemistry. Polymer films of **L** were prepared through oxidative electro polymerization on glassy carbon electrodes in **L** solutions in 0.1 M TBAP in acetonitrile. They were characterized through electrochemistry. The surface of chemically modified electrodes (CMEs) prepared through controlled potential electrolysis (CPE) at variable concentrations, potentials, and electric charges was characterized through scanning electron spectroscopy, atomic force microscopy, and Raman spectroscopy, which confirmed the films’ formation. Electrochemical sensing of the films deposited on these CMEs was tested with respect to heavy metal (HM) ion analysis in aqueous solutions to obtain sensors for HMs. The obtained CMEs presented the best characteristics for the recognition of Pb among the investigated HMs (Cd, Pb, Cu, and Hg). Calibration curves were obtained for the analysis of Pb(II) in aqueous solutions, which allowed for the estimation of a good detection limit of this cation (<10^−8^ M) for non-optimized CMEs. The resulting CMEs show promise for deployment in portable environmental monitoring systems, with implications for public health protection and environmental safety.

## 1. Introduction

The contamination of environmental matrices with heavy metals, such as lead (Pb(II)), cadmium (Cd(II)), mercury (Hg(II)), and copper (Cu(II)), has become a major public health and ecological concern due to their toxicity, persistence, and bioaccumulation [1]. These metals can interfere with biological processes even at low concentrations, necessitating the development of analytical tools that are not only sensitive and selective but also capable of functioning in situ, especially in complex or turbid samples [2,3,4]. Compared to traditional spectroscopic or chromatographic techniques, electrochemical sensors offer numerous advantages, including low cost, portability, rapid response, and minimal sample preparation [5,6,7,8]. In particular, chemically modified electrodes (CMEs) have attracted considerable attention due to their capacity to be molecularly engineered for targeted detection. Incorporation of electron-rich or electron-deficient ligands into sensor surfaces enhances selectivity and signal response for specific analytes [8]. Among these, 1,3,4-thiadiazole derivatives have emerged as efficient chelating ligands for soft heavy metal ions, such as Pb(II) and Cd(II), owing to the presence of sulfur and nitrogen donor atoms in their structure [7,8,9]. These heterocycles have been successfully employed in voltametric platforms for metal ion sensing, especially when integrated into nanostructured films or polymers.

Complementarily, azulene-based monomers provide valuable features, such as dipolar aromaticity, extended π conjugation, and low ionization energy [10,11]. Their electropolymerization on electrode surfaces generates redox-active, conductive films suitable for sensor development. Recent studies have reported on polyazulene- and tetrazole-functionalized azulene materials that can detect Pb(II) and Cd(II) at nanomolar concentrations, demonstrating strong electron transfer behavior and favourable film morphology [12,13].

Data from the literature show that a series of compounds from the diazene class containing heterocycles are compounds of great interest and have been synthesized and studied both due to their stability, easy availability, and lack of toxicity as well as their biological properties (antibacterial, antiviral, anti-inflammatory, or analgesic activity) and their coloring properties. Because 1,3,4-thiadiazole is a stable and easy to obtain heterocycle [14], numerous derivatives of it with various arylazoic substituents have been reported in the literature [15,16]. These compounds have a variety of applications in the technical field, being used as dyes [17,18,19,20,21,22,23] or in the medical field due to their biological properties [24,25] (antimicrobial and antifungal) [26] and cytotoxicity against tumor cells from ascites carcinoma [27].

The introduction of the electron-donating azulen-1-yl group into the diazene heteroaryl structure allows for the preparation of push–pull azo systems, such as az-ulen-1-azopyridines and azopyranilium salts [28], azulen-1-azopyridine 1′-oxides [29], azulen-1-azo-2-thiazoles [30], and azulen-1-azo-2-benzothiazoles [31], if a heterocycle with electron-accepting properties is attached to the N=N double bond [32]. Azo dyes obtained when electron-accepting five-membered rings (furan, pyrrole, thiophene, thiazole, thiadiazole, benzothiazole) are associated with the azulene group show potential nonlinear optical and liquid crystal properties. Compared to classical electropolymerizable monomers, such as pyrrole and thiophene, azulenes exhibit lower ionization energy and higher electronic mobility, making them interesting building blocks for the synthesis of new advanced materials [33].

Răzuș et al. [7] reported the synthesis of 2-(azulen-1-yldiazenyl)-1,3,4-thiadiazole derivatives, including the phenyl-substituted analog of **L**, via diazotization of 1-aminoazulene followed by azo coupling with substituted thiadiazole amines, and demonstrated their ability to electropolymerize into conductive, redox-active films with high affinity for heavy metal ions.

Several derivatives of 2-(azulen-1-yldiazenyl)-1,3,4-thiadiazole have been previously reported, including phenyl- and *p*-methoxyphenyl-substituted analogs [34,35], which exhibit good electroactivity and the ability to form uniform electropolymerized films on glassy carbon electrodes. These films have shown high affinity for soft heavy metal ions, such as Pb(II) and Cd(II), enabling detection at sub-µg/L levels [34,35]. Other structural variants, including thiophene-substituted and vinylene-bridged azulene–thiadiazole systems, have been investigated theoretically for their enhanced π conjugation, reduced band gap, and improved charge transport properties [13,36]. Additionally, halogenated or nitro-substituted derivatives have been explored to modulate electron density and fine-tune metal ion selectivity [37]. Building on these findings, the present study introduces *2-(azulen-1-yldiazenyl)-5-(thiophen-2-yl)-1,3,4-thiadiazole* (**L**) as a novel hybrid monomer designed to combine strong metal binding capacity with efficient charge transport and redox-active electropolymerization.

A particularly relevant compound, 2-(azulen-1-yldiazenyl)-5-phenyl-1,3,4-thiadiazole (**T**), has been previously shown to electropolymerize under controlled potential electrolysis, forming films with increased conductivity and up to sixfold greater electroactive surface area [36]. However, the monomeric structure of **T** lacks the tunability required to optimize film conductivity, redox activity, and metal ion specificity simultaneously.

**Figure 1:**
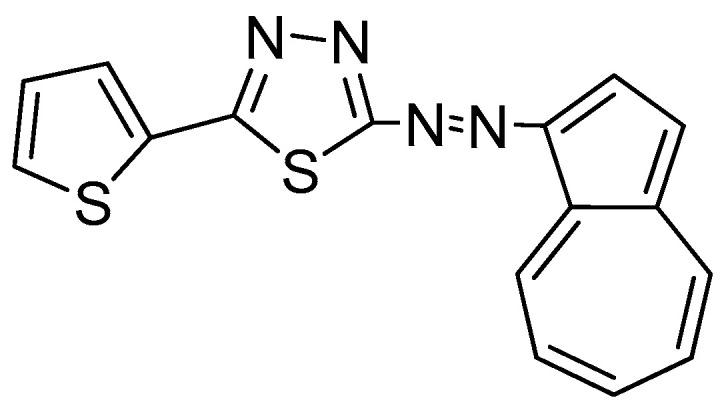
Structure of **L**.

To address these limitations, we tested a novel hybrid monomer, 2-(azulen-1-yldiazenyl)-5-(thiophen-2-yl)-1,3,4-thiadiazole (**L**), with a structure shown in Figure 1, that combines (i) a thiophene group to improve charge transport, (ii) a thiadiazol moiety for metal binding, and (iii) an azulene core to support redox-active electropolymerization. While some analogues, such as T, have been investigated, this is the first report on the electropolymerization, poly**L** film formation, and analytical application of poly**L** in heavy metal sensing.

The present study had two main objectives: (i) to perform the controlled electrodeposition of L films onto glassy carbon electrodes and (ii) to evaluate their performance as voltametric sensors for Cd(II), Pb(II), Cu(II), and Hg(II) detection. The results demonstrate that deposition potential and charge play a decisive role in determining sensor properties. This work introduces **L** as a functional monomer capable of forming stable, redox-active films with high affinity for heavy metal ions.

## 2. Results

2-(Azulen-1-yldiazenyl)-5-(thiophen-2-yl)-1,3,4-thiadiazole (**L**) was synthesized according to the method used in our laboratory [7]. Polymer films of **L** were prepared through electropolymerization in **L** solutions in organic electrolyte, leading to chemically modified electrodes (CMEs). They were examined through electrochemistry. The film attached to their surface was characterized through SEM, AFM, and Raman spectroscopy. Electrochemical sensing of the films that cover the CMEs was tested with respect to heavy metal (HM) ions in aqueous solutions in order to obtain sensors for HMs.

### 2.1. Preparation of Polymer Films and Electrochemical Characterization of CMEs

The electrochemical immobilization of the **L** monomer on the electrode surface was tested either through scanning or controlled potential electrolysis (CPE). CPE was considered the most appropriate method for obtaining reproducible results. CPE was conducted in millimolar solutions of **L** dissolved in acetonitrile (ACN) in the presence of the supporting electrolyte, consisting of tetrabutylamoniumperclorate (TBAP) dissolved in ACN at a concentration of 0.1 M (0.1 M TBAP/ACN). The glassy carbon (GC) electrodes previously cleaned and checked through electrochemistry were polarized at positive potentials. Various charge densities and anodic potentials were applied as a result of the electrochemical study of the ligand detailed in Section 2.5. This procedure, which enabled the formation of polymer films, was based on the results of the study of electrochemical properties of **L** monomer. The preparation of **L**-based chemically modified electrodes (L-CMEs) ensured the anchoring of the ligand’s complexing units onto the electrode surface. The freshly clean GC electrode was polarized through chronoamperometry in a potential step towards positive potentials, and the current was recorded. CPE was stopped after reaching a proposed electric charge. The chronoamperograms were recorded in repeated experiments to check the reproducibility of CMEs’ preparations (Figure S1, Supplementary Materials). The obtained CMEs were characterized through ferrocene redox probe following CPE. The modified electrodes were rinsed with ACN and immersed in a 1 mM ferrocene solution (in 0.1 M TBAP/ACN) to assess film formation.

Table 1 summarizes the preparation parameters for the CMEs obtained via electrochemical oxidation through CPE. The CMEs were characterized using multiple techniques that evidenced film formation, including ferrocene redox probe (Fc), chronoamperometry, SEM, AFM, and heavy metal ions’ (HMs) detection, as seen further.

**Figure 2:**
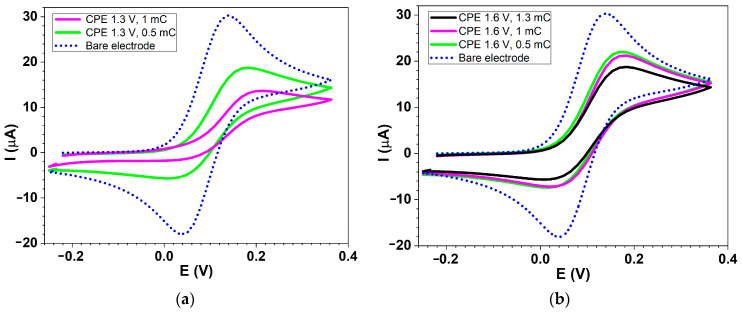
CV curves (0.1 V·s^−1^) recorded in 1 mM ferrocene solution in 0.1 M TBAP/ACN for CMEs prepared through CPE in **L** solution (1.3 mM in 0.1 M TBAP/ACN) at different potentials, (**a**) 1.3 V and (**b**) 1.6 V, using various electropolymerization charges (solid lines) vs. bare electrode (dashed lines).

Figure 2 shows several comparative CV curves recorded in ferrocene solution for CMEs prepared through CPE in **L** solution at two potentials using various electropolymerization charges. Solid lines represent the CV curves for CMEs, and the dashed line corresponds to the ferrocene signal on the bare electrode without film.

### 2.2. SEM Characterization of CMEs

**Figure 3:**
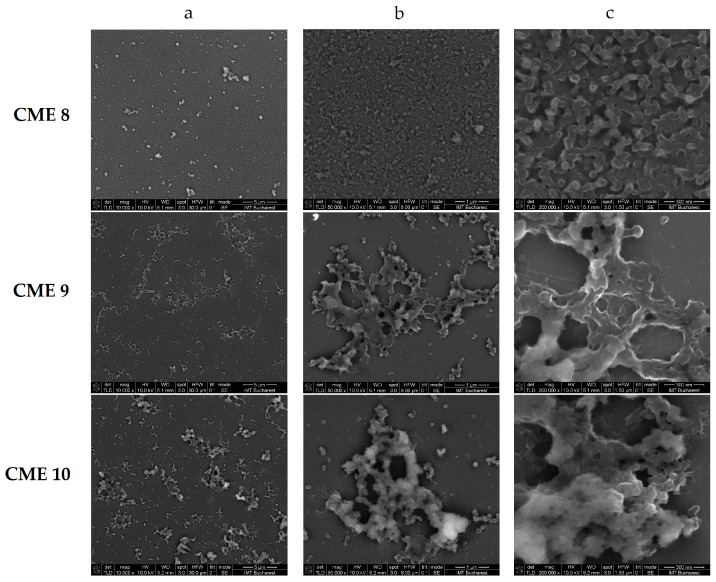
SEM images at different magnifications, 10,000 (**a**), 50,000 (**b**), and 100,000 (**c**), for CME 8 (CPE at +0.9 V, 4 mC), CME 9 (CPE at +1.3 V, 4 mC), and CME 10 (CPE at +1.3 V, 14 mC).

The surfaces of CMEs based on **L** (**L**-CMEs) were investigated using SEM to determine the characteristics of the films deposited on GC6 discs (diameter 6 mm) obtained at different potentials and electropolymerization charges. Several SEM micrographs are shown in Figure 3 for different charges and potentials.

### 2.3. AFM Characterization of CMEs

The surfaces of CMEs based on **L** (**L**-CMEs) were investigated using AFM for the films deposited on GC6 discs (diameter 6 mm). Several AFM images are shown in Table 2 for different electropolymerization potentials and charges. The thickness of the films deposited through CPE was measured using a technique involving scraping (mechanical removal) the film with the AFM tip, followed by scanning across an area that includes both the scraped region and the adjacent intact surface. While scraping alters the tip’s geometry and inevitably degrades subsequent image quality, it does not affect the key measurement, namely, the step height. The results obtained for three films (CME 8, CME 9, and CME 10) are depicted in Table 2.

### 2.4. Raman Characterization of CMEs

**Figure 4:**
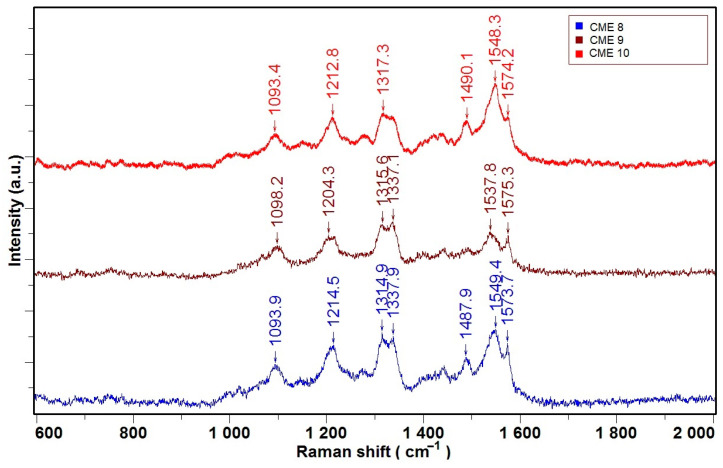
Raman spectra acquired for CMEs obtained at different potentials and charges; the films were prepared through CPE at +0.9 V, 4 mC (CME 8), +1.3 V, 4 mC (CME 9), and +1.3 V, 14 mC (CME 10).

Figure 4 shows Raman spectra for films obtained at different potentials and charges on CME 8, CME 9, and CME 10.

### 2.5. Electrochemical Sensing of Heavy Metals (HMs) Using CMEs

Electrochemical investigations of CMEs were directed to find the conditions for preparing complexing films (see Section 2.1). The variables were the monomer concentration, the applied potential, and the involved electric charge.

**Figure 5:**
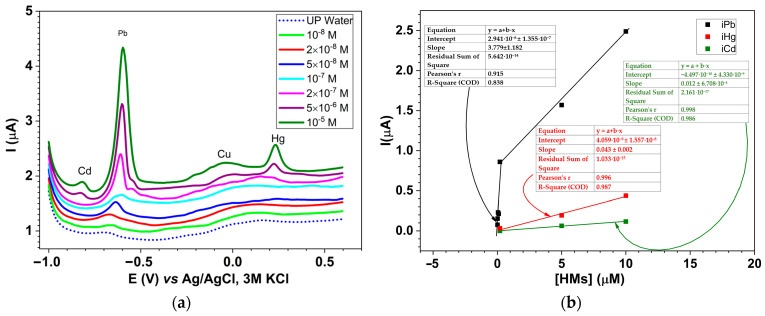
Stripping curves (0.01 V/s) recorded in 0.1 M acetate buffer (pH 4.5) after 15 min of accumulation in solutions containing equal concentrations of Cd(II), Pb(II), Cu(II), and Hg(II) in water (**a**); calibration curves for Pb(II), Hg(II), and Cd(II) on CMEs according to (**a**,**b**); the modified electrodes obtained through CPE at +1.3 V using an electrical charge of 1 mC in 1 mM L solution in 0.1 M TBAP/ACN.

The chemically modified electrodes based on **L** (**L**-CMEs) were evaluated with respect to their ability to recognize the heavy metal (HM) ions from aqueous solutions containing Cd(II), Pb(II), Cu(II), and Hg(II) in equal concentrations ranging from 10^−8^ M to 10^−5^ M. Taking into account the results obtained during the study of CMEs’ preparation at different electropolymerization potentials and charges, the CMEs’ preparation conditions were chosen. The CMEs used for HM analysis were prepared via CPE at +1.3 V, 1 mC in a 1 mM solution of **L** in 0.1 M TBAP/ACN. We selected these parameters as proof of concept to determine which cations under investigation respond, which give the best response, etc. After synthesis, the electrodes were rinsed with acetonitrile, *conditioned*, and *overoxidized* in a 0.1 M acetate buffer (pH 4.5) according to a procedure previously established [38]. Each conditioned CME was then rinsed with ultrapure water and immersed in an aqueous solution containing the same molar concentration of Cd(II), Pb(II), Cu(II), and Hg(II) ions under magnetic stirring. The amount of each metal ion complexed within the film was quantified using anodic stripping voltammetry (ASV) in acetate buffer, employing software for differential pulse voltammetry (DPV). After a 3 min pre-concentration step at −1.2 V to reduce all metal ions, DPV stripping was performed from −1.2 V to +0.5 V. The resulting DPV curves (Figure 5a) confirmed the presence of HM ions in the **L**-CME films, with characteristic stripping peaks noticed at −0.82 V (Cd), −0.61 V (Pb), −0.055 V (Cu), and +0.24 V (Hg). The peaks are distinguished from the background noise (the peak height for each cation has been calculated as the difference between the current of the peak and the corresponding current of the background at the peak potential). Figure 5b illustrates the correlation between stripping peak current intensities for the Pb(II) ion and their concentration in the tested solutions. It also contains the DPV dependencies corresponding to the DPV peaks of Hg(II) and Cd(II) (red and green lines, respectively), together with the equations of the straight lines (written in the same colors).

Apart from the points corresponding to the lowest concentration, which are linearly corelated (Pearson’s r = 0.915), the points corresponding to the highest investigated concentrations were plotted on the same graph, which was interrupted on the concentration axis.

### 2.6. Investigation of the Ligand’s Electrochemical Properties

The electrochemical characterization of **L** was performed on GC electrodes using CV, DPV, and RDE methods. Initially, voltametric recordings were performed in the supporting electrolyte (0.1 M TBAP/ACN) to establish the baseline oxidation and reduction curves. These reference curves, obtained at the start of each experiment in the absence of the ligand, are shown as dashed lines in the figures. Subsequent measurements were conducted using **L** solutions in 0.1 M TBAP/ACN at different concentrations. The DPV recordings were used as a reference to identify the peaks in the other techniques. The main peaks seen in all voltammograms were labeled in an order corresponding to the DPV peak potentials as they appeared in the recordings.

#### 2.6.1. Electrochemical Study of the Ligand Through DPV

**Figure 6:**
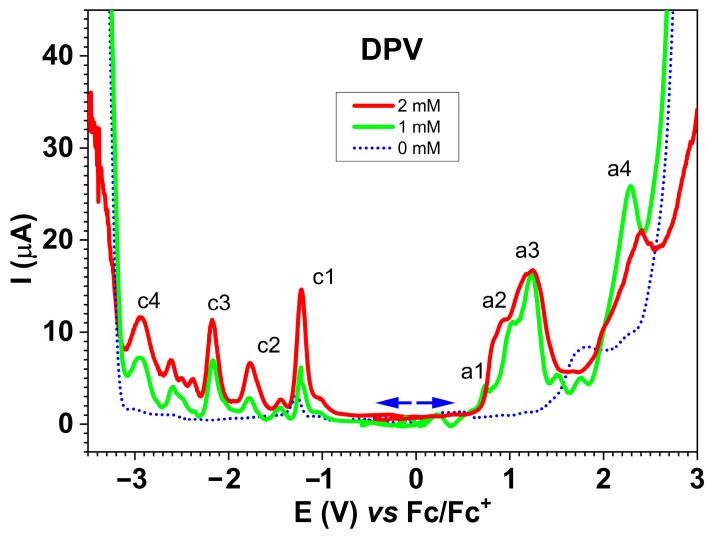
DPV curves for different concentrations of **L** in 0.1 M TBAP/CH_3_CN.

Oxidation and reduction curves were recorded using the DPV technique at various concentrations of **L** (0–2 mM) in 0.1 M TBAP/ACN, starting from the stationary potential, as seen in Figure 6. The starting points for oxidation and reduction are indicated by arrows (→ and ←, respectively). The main oxidation (a1–a4) and reduction (c1–c4) peaks were identified. The peak currents increased with **L** concentration.

#### 2.6.2. Electrochemical Study of the Ligand Through CV

**Figure 7:**
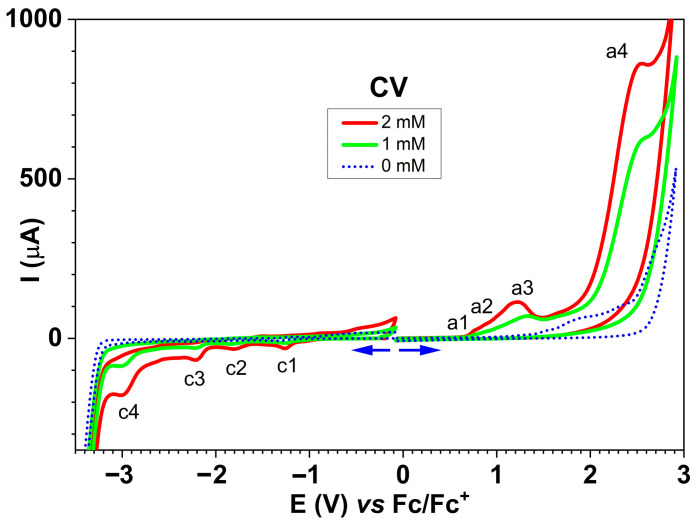
CV curves for different concentrations of **L** in 0.1 M TBAP/CH_3_CN.

CV curves (Figure 7) show the oxidation peaks (a1–a4) and the reduction peaks (c1–c4) starting from the stationary potential. The anodic and cathodic peak currents increased with **L** concentration.

**Figure 8:**
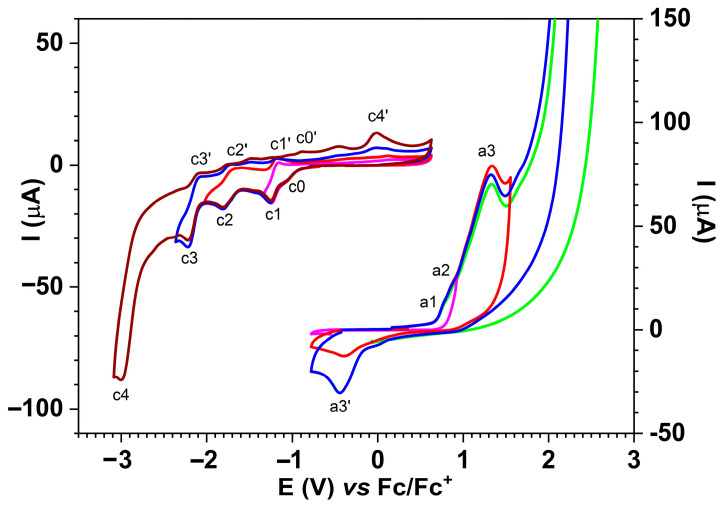
Curves for **L** in TBAP/CH_3_CN 0.1 M at 1 mM concentration obtained through CV (0.1 V/s) on different anodic and cathodic scan domains.

The CV curves were initially recorded on different anodic scan domains (Figure 8), and the peaks were denoted in agreement with the DPV peaks in Figure 7.

**Figure 9:**
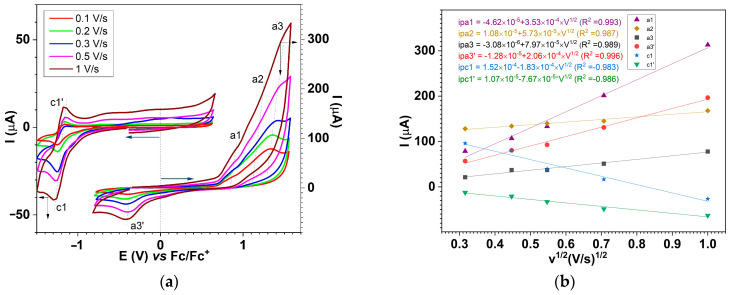
CV curves in the cathodic and anodic potential ranges at various scan rates for [**L**] = 1 mM in 0.1 M TBAP/ACN (**a**); linear dependences of a1, a2, a3, a3′, c1, and c1′ peak currents on the square root of the scan rate in V/s (**b**).

Figure 8 shows the CV curves obtained at different scan rates up to potentials in the first cathode peak c1 range (Figure 9a, up) and towards anodic potentials (Figure 9a, down). The starting potentials of the sweeps are marked by arrows that also indicate the direction of the sweep. The currents increase with the scanning rate. The representation of the currents as a function of the scanning rate radical leads to straight lines, whose equations have been calculated (Figure 9b).

The values of the peaks’ potentials for CV and DPV curves are given in Table 3, as well as the characteristics of the peaks resulting from CV curves.

#### 2.6.3. Electrochemical Study of the Ligand Through RDE

**Figure 10:**
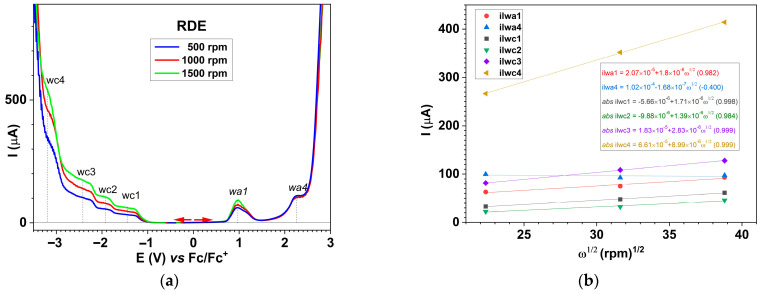
RDE curves in anodic and cathodic scans at different rotation rates on GC electrode recorded in a solution of 1 mM L in 0.1 M TBAP/ACN (**a**), and the linear dependences of the limiting currents on the square root of the electrode rotation rate ω^1/2^ (**b**).

Oxidation and reduction curves were recorded using the RDE technique at different rotation rates (500–1500 rpm) for **L** in 0.1 M TBAP/ACN, starting from the stationary potential, as seen in Figure 10. The main oxidation and reduction (w1–w4) waves were identified corresponding to the peaks obtained in DPV and CV curves (see Figure S2). The currents of the anodic and cathodic waves increased with the electrode rotation rate in a different manner, as will be discussed further in the Section 3.

## 3. Discussion

The conditions for obtaining poly-**L** films established based on the analysis of the results obtained from the electrochemical characterization of ligand **L** have been exploited to establish a good procedure for the preparation of CMEs. The results of their characterization are discussed below and connected to the discussion of ligand electrochemical properties.

### Polymer Film Preparations and Electrochemical Characterization

The electrochemical immobilization of the **L** monomer on the electrode surface was achieved via controlled potential electrolysis (CPE), which was considered the most appropriate method for obtaining reproducible results (Figure S1). Table 1 underlines the methods used to characterized the obtained CMEs: ferrocene redox probe, chronoamperometry, SEM, AFM, and heavy metal ion detection. CMEs were obtained via electrochemical oxidation through CPE in different conditions of preparation. All techniques evidenced film formation.

The characterization of several obtained CMEs through ferrocene redox probe in ferrocene solutions given in Figure 2 is resumed in Table 4. They are the main electrochemical parameters of the ferrocene/ferrocenium (Fc/Fc^+^) redox couple peaks, which are derived from these CV curves. These include the anodic peak potential (Epa), the cathodic peak potential (Epc), the peak-to-peak separation (ΔEp), the formal potential (Ef), the anodic peak current (ipa), and the cathodic peak current (ipc) measured from the 0 line for each glassy carbon-based chemically modified electrode (**L**-CME). Table 4 shows that ipa values recorded on CMEs diminished with respect to the ferrocene current on bare electrodes (3.025 × 10^−5^ A). This indicates the covering of the electrode with films that partially blocked the electrode surface. Variations in the ferrocene signal parameters can be correlated with the differences in film coverage and properties. The increase of the applied potential in CPE from 1.3 V to 1.6 V leads to the increase of ipa from 1.887 × 10^−5^ A to 2.215 × 10^−5^ A (lines 2 and 4 in Table 4). Similarly, this increase of the applied potential leads to the increase of ipa from 1.356 × 10^−5^ A to 2.124 × 10^−5^ A (3 and 5 in Table 4). The increase of the electropolymerization charge leads to the decrease of the ipa ferrocene current, which shows the formation of a thicker film. For instance, the increase of charge from 0.5 mC to 1.3 mC in CPE performed at +1.6 V leads to the decrease of ipa from 2.215 × 10^−5^ A to 1.881 × 10^−5^ A (lines 4–6 in Table 4). The formation of films leads to the change of Epa to more positive potentials, while Epc is not so much changed. Consequently, ΔEp increases with the involved charge in electropolymerization.

The analysis of the data in Table 4 shows that the ferrocene signals on the electrodes covered with films decrease in intensity with the electropolymerization charge. The difference between the peak potentials ΔEp increases with the charge. Calibration of the film’s thickness (proportional to the electropolymerization charge) could be possible using this parameter.

Figure 3 clearly illustrates the progressive changes of the film structure with potential and charge (clear to all magnifications). At an applied potential of +0.9 V and a total charge of 4 mC (CME 8), the SEM analysis reveals the presence of small, well-defined granular structures that are uniformly distributed across the surface. The morphology of these structures appears to be relatively smooth, which suggests the formation of a consistent, uniform film with good coverage. At lower voltages and charges, it leads to a thin, evenly distributed film with minimal aggregation.

By increasing the potential to 1.3 V while maintaining the charge at 4 mC (CME 9), a film with larger agglomerations or aggregates was obtained. These agglomerations become interconnected, forming network-like structures, with a significant increase in surface roughness and porosity. This indicates the development of a more complex surface morphology, which suggests an increase in the electroactive surface area at this increased potential.

When increasing the charge (to 14 mC at +1.3 V), it results in a film (CME 10) with denser, larger aggregates that are significantly more pronounced than in CME 9 (4 mC at +1.3 V), which has complex, densely packed morphologies, indicating a thick and highly structured film. A higher charge at the same voltage leads to more extensive film growth, resulting in dense and complex surface morphologies. This morphology is compatible with the formation of a thicker film with an enhanced surface area (Table 5).

The morphological differences noticed through SEM were further investigated using atomic force microscopy (AFM). The AFM images (Table 2) show that apart from the large, evenly sized aggregates (hundreds of nanometers) arising on all sample surfaces and visible also in the SEM images (Figure 3), the underlying deposited films are continuous and smooth. It was this feature that enabled the ready detection and measurement of even the 10 nm ultrathin film in sample CME 9. For sample CME 10, this procedure was not able to completely remove the film from the scrapped area, which could be attributed to greater film adherence for this sample (Table 5).

The AFM microscope proprietary image processing software (Image Analysis(C)) enables refining the achieved results to estimate the average equivalent thickness (*teff*)—that is, the thickness that would result if the material from surface aggregates was uniformly distributed across the film surface. The estimated equivalent thicknesses are

- CME 8: *teff* = 161 nm (an extra 6 nm compared with the underlying 155 nm);
- CME 9: *teff* = 73 nm (an extra 63 nm compared with the underlying 10 nm);
- CME 10: *teff* = 39 nm (an extra 13 nm compared with the underlying 26 nm).

However, given the restricted scan size and limited data, these values provide only an approximate indication. Nevertheless, they support the interpretation—suggested by AFM images—that the initially smooth base film may grow in thickness and roughness via the incorporation of surface aggregates. As such, the contribution of aggregates to the average equivalent thickness teff is highest for the thinnest film (CME 9) and decreases with increasing film thickness, such that in the thickest film (CME 8), the aggregates appear to be fully integrated into the film.

The identified bands in Raman spectra of CME 8–CME 10 (Figure 4) most likely correspond to the following vibration assignments [39,40,41,42]:

- 1093 cm^−1^—C—H bonds;
- 1212 cm^−1^—C—H bonds (from vinylene group);
- 1314 cm^−1^, 1337 cm^−1^—C—C bonds (possible in the range of 1202–1367 cm^−1^);
- 1482–1509 cm^−1^—(C–C=C) group;
- 1490 cm^−1^—C=C—thiophene group;
- 1548 cm^−1^, 1574 cm^−1^—C=C bonds from aromatic/olefinic groups.

All Raman spectra of the three CME films exhibit all characteristic peaks in the range of 1090 to 1575 cm^−1^, indicative of aromatic and carbon-based structures. Peaks around 1317–1337 cm^−1^ and 1574–1575 cm^−1^ correspond to the D and G bands, respectively, which are typical of disordered and graphitic carbon materials, such as graphene, carbon nanotubes, and graphite [43]. The presence of these bands suggests varying degrees of structural order and conjugation among the samples. Additional peaks near 1212–1214 cm^−1^ and 1487–1490 cm^−1^ are consistent with C–N and C=C stretching vibrations found in polyaniline and other aromatic polymers [44]. The red spectrum (CME 10) shows the most intense and well-defined peaks, indicating a higher degree of conjugation or graphitic character. In our case, the ligand’s structure ensures a high degree of conjugation, and that is one of the reasons for its synthesis in view of obtaining highly conjugated polymer films. These spectral features align with known Raman profiles of carbon-based nanomaterials and conjugated polymers, confirming the successful modification and structural diversity of the chemically modified electrodes [45].

The SEM images revealed distinct differences in the surface morphology of CME 8–CME 10 films, with CME 10 exhibiting a more compact and homogeneous structure, suggesting enhanced film uniformity and coverage. AFM measurements corroborated these observations by quantifying higher surface roughness parameters for CME 8 and CME 9 compared to CME 10, indicating that the latter possessed a smoother topography and potentially stronger substrate adhesion. The incomplete removal of the CME 10 film during the scratch test further supports its superior adhesion properties.

Raman spectroscopy complemented the morphological analyses by confirming the chemical composition and structural features of the films. The spectra for all samples displayed characteristic bands attributable to C–H, C–C, and C=C stretching vibrations, with variations in peak intensity and position reflecting differences in molecular ordering and conjugation. Notably, the more pronounced and sharper C=C-related bands in CME 10 may be indicative of a higher degree of π conjugation and structural ordering, which could contribute to the enhanced adhesion and uniformity observed in SEM and AFM analyses.

Electrochemical investigations for HM recognition on **L**-CMEs revealed a higher signal for Pb (II) among all HMs’ investigated cations (Cd(II), Pb(II), Cu(II), and Hg(II)). The amount of each metal ion complexed within the film was quantified using anodic stripping voltammetry (ASV) employing differential pulse voltammetry (DPV). The resulting DPV curves confirmed the presence of HM ions in the **L**-CME films. Among all of the tested cations, lead exhibited the strongest analytical signal (at −0.67 V), with clear detection achievable in accumulation solutions at concentrations above 10^−8^ M (Figure 5b). In contrast, cadmium and mercury produced only weak DPV signals (at −0.82 V and 0.22 V, respectively), which became noticeable only at concentrations exceeding 5 × 10^−6^ M and 2 × 10^−7^ M, respectively. The calibration curve for Pb(II) on CMEs given in Figure 5b indicates linear behavior followed by a plateau. From the linear part on the calibration curve, a good detection limit under 10^−8^ M for Pb(II) can be estimated. Limits of the detection value for each metal were estimated from Table S3 to be 10^−8^ M, 2 × 10^−7^ M, and 5 × 10^−6^ M for Pb(II), Hg(II), and Cd(II), respectively. For Cu (II), there is no signal to certify its complexation by this ligand. However, the preparation conditions for CMEs could be optimized, leading to improvements of these detection limits, which are useful for several specific applications.

Concerning the electrochemical study of the ligand, the DPV curves highlighted the main processes that occur during the electrochemical oxidation of the ligand (when the sweep occurs towards anodic potentials) or during its electrochemical reduction (when the sweep occurs towards cathodic potentials).

The conditions for obtaining poly-**L** films were established based on the analysis of the results obtained from the electrochemical characterization of ligand **L**. The DPV curves highlighted the main processes that occur during the electrochemical oxidation of the ligand (when the sweep occurs from the equilibrium potential towards more positive potentials) or during its electrochemical reduction (when the sweep occurs from the equilibrium towards more negative potentials).

DPV curves (Figure 6) exhibited four main oxidation peaks (a1–a4) and four main reduction peaks (c1–c4). Their potentials are given in Table 3. They were attributed to electrooxidation and electroreduction processes of the ligand as the peak currents increase (in absolute value) with **L** concentration. The process a4 occurs superimposed on the oxidation processes of the supporting electrolyte, which begin at approximately 1.3 V.

In analyzing the cathodic curves in Figure 6 and Figure S3 (Supplementary Materials), regarding the influence of concentration on the currents in the DPV curves, it can be observed that the currents are higher in all processes attributed to the ligand. For potentials more negative than −3.2 V, reduction of the supporting electrolyte occurs. At a concentration of 2 mM, this process is greatly diminished due to the irreversible process c4. c4 can be attributed to the formation of films through the electroreduction of **L**, which blocks the electrode surface, as in the case of other azulene compounds (azulene can form films through both electrooxidation and electroreduction; in the latter case, the potential values are higher in absolute values) (azulene is more difficult to reduce than to electrooxidize).

The CV curves recorded for different concentrations of **L** (Figure 7) confirmed the processes highlighted by DPV (Figure S3). The CV peak potentials (shown in Table 3) are consistent with the peak potentials observed in DPV. By comparing the CV and DVP curves (Figure S1), the similarities between the processes highlighted by the two methods can be better observed. The peak potentials appear to be shifted towards higher absolute values for the anodic and cathodic peaks in CV compared to the peak potentials established (more precisely) by DPV. The parallel presentation of the processes by CV and DPV (Figure S3) shows the concordances between the main processes highlighted by the two methods (dotted lines in Figure S3 connect the two processes in CV and DPV).

On the curves obtained through CV (Figure 8) for **L** (at 1 mM concentration) on different anodic and cathodic scan domains, the main peaks are obtained in the forward scan (noted in the order of their appearance in the scan) and the corresponding peaks are obtained in the reverse scan (marked with the prime symbol). It can be observed that peaks c1, c2, and c3 correspond to peaks c1′, c2′, and c3′ in the return scans, which led us to consider them as originating from reversible processes, while peaks a1-a4 and c4 do not have peaks located at close potentials on the return curve and were therefore considered irreversible (Table 3).

The evolution of the main peaks can be studied by correlating the CV curves obtained at different scan ranges (Figure 8) with those obtained at different scan rates (Figure 9). Thus, the c1 process appears to be reversible both from the examination of the CV curves shown in Figure 8 (up) and the curves obtained at different scan rates (Figure 9, up). The peak current c1 increases linearly in absolute value with the square root of the scan rate with a slope of 1.83 × 10^−4^ A·V^−1/2^·s^1/2^ (Figure 9b), and the corresponding peak in the return scan (c1′) also has increasing currents in absolute values. The slope of peak c1′ is smaller (7.67 × 10^−5^ A·V^−1/2^·s^1/2^) than that of the c1 peak, but the currents were measured relative to the value 0 of the current and not relative to the baseline of the peak in the return scan. The correlation coefficient for the line ipc1 vs. v^1/2^ (0.983) is similar to that for the line ipc1′ vs. v^1/2^ (0.986). Some errors connected to overlapping of the parasitic process (whose peaks are denoted by c0 and c0′ in Figure 8) are possible. This last process is due to traces of molecular oxygen in the supporting electrolyte (which was previously degassed with argon), which is reduced to the anion radical (O_2_/O_2_^−·^). Analyzing the slopes (in A·V^−1/2^·s^1/2^) for the current dependencies for processes a1, a2, and a3 from Figure 9b, it can be seen that they vary in the order a1 > a2 < a3 (~35.3 × 10^−5^ > 5.73 × 10^−5^ < 7.97 × 10^−5^), which is consistent with the assignments made for these processes. Process a1 corresponds to an electron transfer (E), while processes a2 and a3 correspond to complex mechanisms (ECE) related to film formation. The differences between the slopes for a2 and a3 reflect a more intense polymerization at the potential of process a2 and justify the choice of 1.3 V as the potential for testing the new **L**-based CME as a proof of concept. Comparing processes a1 and c1 of ligand oxidation and reduction, it can be stated that the number of electrons involved in the first oxidation step is greater than that in the first reduction step because the slope of a1 (3.53 × 10^−4^ A·V^−1/2^·s^1/2^) is higher than the absolute value of the slope for c1 (1.83 × 10^−4^ A·V^−1/2^·s^1/2^). The peaks are better defined only for the first oxidation process a1, while for a2 and a3, which take place at potentials close to the a1 peak, the currents read are total currents. The slope of the current dependence on the square root of the scan rate for a2 (Figure 9b) is smaller (5.73 × 10^−5^ A·V^−1/2^·s^1/2^) than the slope of a1 (35.3 × 10^−5^ A·V^−1/2^·s^1/2^) because insulating films are formed in the a2 process that cover the electrode. Considering a number of two electrons/molecules involved in the first anodic oxidation process a1 of **L** to the dication (**L**^2+^), based on the Randles–Ševčík equation [13], the **L**’s diffusion coefficient of 8.6 × 10^−5^ cm^2^·s^−1^ was obtained.

The oxidation and reduction curves obtained using the RDE technique at different electrode rotation rates recorded in the **L** solution, shown in Figure 9, show that the oxidation processes are more complex than the reduction processes in terms of electron transfer. The oxidations proceed practically through complex three-step mechanisms: electrochemical (E)–chemical (C)–electrochemical (E). The chemical (C) step is the one that leads to chain propagation in the case of electropolymerization. CV cannot clearly show film formation because the scanning is quite fast. However, using the DPV technique, which was conducted at the same scan rate as the RDE technique, there is good agreement between the decrease in currents in RDE at the potentials of peaks a2 and a4, where we assumed film formation occurs (Figure S2).

Cathodic RDE processes are successive regular reduction waves (w1–w4) and correspond with the cathodic DPV and CV processes (Figures S2 and S3). These waves have currents that increase with the square root of the electrode rotation rate (Table S1). The equations of these dependences are given in Table S2. From the ratio between their slopes, the ratio between the number of electrons involved in these processes has been estimated to be nc1/nc2/nc3/nc4 = 1/1/3/9. Figure 10b shows that all lines expressing the dependencies of the limiting currents as a function of the square root of the electrode rotation rate have good correlation coefficients (for ilimwa1, ilimwc1–4), except for ilimwa4, which has a very weak correlation coefficient compared to the other dependencies on the rotation rate. This behavior is due to the fact that at this potential (characteristic for the a4 peak), a film is formed. The film covers the electrode and leads to a decrease in current, as shown by the DPV and RDE curves. Thus, in Figure 6, the peak DPV current for a4 at 2 mM is lower than at 1 mM (which is not the case for the other processes), a fact that can be explained by the fact that the polymerization process is more intense at higher concentrations (2 mM). Also, in Figure 10a, the RDE limit current for wa4 does not depend on the electrode rotation rate, which can only be explained by the hypothesis that a film has formed, and in Figure 10b it was observed that there is no linear correlation for wa4 with the square root of the electrode rotation rate (ω^1/2^).

The anodic RDE process wa1 is not seen as an oxidation wave but as a peak with a maximum current that increase with the square root of the rotation rate with the slope of 1.80 × 10^−6^ A·rpm^−1/2^ (Table S2). Then, a sudden decrease of the anodic current to the background values occurs. This behavior indicates the formation of polymer films through ligand electrooxidation at the potential of this wave, as in the case of other azulene ligands [34]. In the case of **L,** the decrease appears at around +0.9 V (Figure 8), a potential that corresponds to the a2 process in DPV (Table S1). After this potential, the current decreases to the baseline until around +1.5 V, and then it increases again. Further oxidation, seen as a wave (wa4), has a limiting current about 10^−6^ A (Table S1) higher than ilwa1, which does not depend on the rotation rate of RDE. The last characteristic involves the formation in the range of potentials of the a4 process of a more compact film than in wa1. The investigation through SEM and AFM of the film obtained at +0.9 V and +1.3 V (CME 8 and CME 10, respectively) revealed greater porosity and aggregation of the film prepared at wa4 wave potentials, as discussed before (Table 5).

## 4. Materials and Methods

The ligand 2-(azulen-1-yldiazenyl)-5-(thiophen-2-yl)-1,3,4-thiadiazole (**L**) was synthesized according to the literature [7]. It was characterized by its crystalline state, melting point (m.p.), UV-Vis wavelength (λ) and the corresponding extinction coefficient on the logarithmic scale (log ε) from the spectra recorded in methanol (MeOH), chemical shifts for ^1^H-NMR and ^13^C-NMR peaks from spectra recorded in deuterated chloroform—singlet (s), doublet (d), triplet (t), and their coupling constant (J), IR wavelengths and their intensity—as weak (w), medium (m), strong (s), and very strong (vs), mass spectrum (MS), and elemental analysis calculated and found for C_16_H_10_N_4_S_2_. The main characteristics are given in the Supplementary Materials.

The acetonitrile (CH_3_CN) solvent was procured from Sigma-Aldrich (electronic grade, 99.999% trace metals; Hamburg, Germany), and the supporting electrolyte, tetrabutylammonium perchlorate (TBAP) (analytical purity ≥ 99.0%), was obtained from Fluka (Munich, Germany). For the purpose of analytical study of HMs, test solutions were prepared using high-purity metal salts; mercury (II) acetate (≥98%), cadmium nitrate tetrahydrate (≥98%), copper (II) acetate monohydrate (≥98%), and lead (II) nitrate (≥99.5%) were all sourced from Fluka, Munich, Germany. Stock solutions of these salts were initially prepared at a concentration of 10^−2^ M, and subsequent dilutions were carried out to obtain test solutions with desired concentrations.

Electrochemical study of the ligand (**L**) and the preparation of the chemically modified electrodes with **L** were carried out using a PGSTAT 12 AUTOLAB potentiostat (Utrecht, The Netherlands) configured with a standard three-electrode setup [36]. For the electrochemical study, the working electrode was a glassy carbon (GC) disc (3 mm diameter, Metrohm, Herisau, Switzerland), either unmodified or modified. A platinum wire served as the counter electrode. The reference electrode was Ag/10 mM AgNO_3_ in 0.1 M TBAP/CH_3_CN (for non-aqueous media).

Electrochemical investigations were conducted using cyclic voltammetry (CV), rotating disc electrode (RDE) voltammetry, and differential pulse voltammetry (DPV). CV measurements were typically performed at a scan rate of 100 mV·s^−1^. DPV experiments were carried out at a scan rate of 10 mV·s^−1^, employing a pulse amplitude of 25 mV and a step time of 0.2 s. RDE studies were conducted at the scan rate of 10 mV·s^−1^, with electrode rotation speeds ranging from 500 to 1500 rpm.

Initial voltametric recordings were performed in the supporting electrolyte (0.1 M TBAP/ACN) to establish the baseline oxidation and reduction curves. These reference curves were obtained at the start of each experiment in the absence of the ligand, and they are shown as dashed lines in the figures. Subsequent measurements were conducted using **L** solutions in 0.1 M TBAP/ACN at different concentrations. The peaks seen in these voltammograms were labeled in the order in which they appeared in the DPV recordings. The DPV recordings were used as a reference to identify the peaks in the other techniques. To facilitate comparison of current intensities, cathodic currents were occasionally presented using their absolute values. All electrochemical characterizations of the ligand were performed in acetonitrile under an argon atmosphere. The measured potentials were referenced to the ferrocene/ferrocenium (Fc/Fc^+^) redox couple.

Electrode modifications were performed via controlled potential electrolysis (CPE) in acetonitrile solutions containing millimolar concentrations of ligand **L** and 0.1 M tetrabutylammonium perchlorate (TBAP) as supporting electrolyte. The working electrodes were GC discs with either 3 mm diameter (GC3) or 6 mm diameter (GC6).

Chemically modified electrodes (CMEs) were evaluated for their sensitivity to heavy metal ions in a 0.1 M acetate buffer solution (pH 4.5), following the methodology outlined in references [35]. The working electrode was the CME based on **L**, the counter electrode was platinum, and the reference electrode was Ag/AgCl in 3 M KCl (for aqueous systems). Stock solutions of HMs were prepared, followed by successive dilution with deionized water to obtain the desired concentration of each HM.

The CMEs were conditioned [38] in 15 CV scans from −0.9 V to +0.6 V (0.1 V/s), followed by overoxidation through 15 CV cycles from −0.2 V to +1.5 V (0.1 V/s). To accumulate the HM ions in the film deposited on CMEs, each conditioned CME was rinsed with ultrapure water and immersed in an aqueous solution containing HM ions in a given concentration (starting from the lowest concentrations we would like to achieve using a sensor based on chemically modified electrodes cu **L**) under magnetic stirring for 15 min. Then, the modified electrode, which had accumulated the HM ions, was rinsed again with ultrapure water to eliminate the HM solution on its surface. The amount of each metal ion complexed within the film was quantified using anodic stripping voltammetry (ASV), employing differential pulse voltammetry (DPV) via potentiostat software. After a 3 min pre-concentration step in 0.1 M acetate buffer at −1.2 V, applied to reduce all heavy metal ions, DPV stripping was performed from −1.2 V to +0.5 V (scan rate: 0.01 V/s; pulse height: 0.025 V; step time: 0.2 s).

The surface analyses of **L**-CMEs used atomic force microscopy (AFM), scanning electron microscopy (SEM), and Raman spectroscopy. For this analysis, CMEs were prepared on 8 mm diameter glassy carbon (GC) discs supplied by OrigaLys (Les Verchères, France) with an exposed surface area of 6 mm (GC6). The charge applied during electropolymerization was adjusted proportionally to the exposed surface area.

SEM characterization of the films was carried out using a Field Emission Scanning Electron Microscope (FEG-SEM), specifically, the Nova NanoSEM 630 model manufactured by the FEI Company (Hillsboro, OR, USA).

The AFM measurements were performed using an AFM system Ntegra Aura (Nt-MDT Spectrum Instruments, Moscow, Russia), operated in Contact mode for the scraping step and Semicontact mode for the measurement step, by employing Point-ProbeR Plus (Nanosensors, Neuchatel, Switzerland) medium-soft cantilevers. AFM image processing was carried out using AFM microscope proprietary image processing software (Image Analysis(C) 3.5.0.18694).

Raman spectra were acquired using a high-resolution LabRAM HR 800 spectrometer (Horiba Jobin Yvon, France). Raman spectra were collected using a 633 nm HeNe laser, with the laser power attenuated by a factor of 10^3^ to minimize sample photodegradation and ensure an optimal signal-to-noise ratio.

All experiments were performed at room temperature (25 °C).

## 5. Conclusions

This work introduces 2-(azulen-1-yldiazenyl)-5-(thiophen-2-yl)-1,3,4-thiadiazole (**L**) as a functional monomer capable of forming stable, redox-active films with high affinity for the Pb(II) ion. The electrochemical characterization of the monomer **L** was performed in acetonitrile solutions in the presence of tetrabuylammonium perchlorate on GC electrodes using CV, DPV, and RDE methods. All methods revealed pronounced asymmetry between the oxidation and reduction curves, indicating mainly reversible electrochemical processes occurring at cathodic potentials and irreversible processes with film formation at anodic potentials. The three methods lead to concordant results regarding the formation of films through electropolymerization of the ligand **L**.

The conclusions drawn from examining the films obtained under different electropolymerization conditions through surface analyses performed using SEM, AFM, and Raman spectroscopy allowed for evaluation of the porosity, aggregation, and adhesion of the films obtained through electropolymerization, which is particularly useful for specific applications of these films (optical or electrochemical).

The morphological differences observed through SEM and subsequently investigated using AFM confirm that higher deposition rates at high potentials favor the formation of nanostructured surfaces with increased roughness. These were tested for the detection of heavy metals (Cd (II), Pb (II), Cu(II), and Hg (II)) for analytical applications based on modified electrodes. Among the monitored metals, the best signal on the unoptimized modified electrodes was obtained for Pb, whose detection limit was below 10^−8^ M, which proves an electrochemical performance that can be improved. The detection limits for Hg(II) and Cd(II) were estimated at 2 × 10^−7^ M and 5 × 10^−6^ M, respectively. No clear signal for Cu(II) was obtained. The resulting CME ensures a high concentration of complexing sites, which leads to a measurable signal using electrochemical methods, even from a very dilute solution (10^−8^ mol/L). They show promise for deployment in portable environmental monitoring systems, with implications for public health protection and environmental safety. Other experiments are in progress to determine the influence of preparation conditions on sensor response in terms of sensitivity and selectivity.

## Figures and Tables

**Table 1:** CME characterization methods by ferrocene redox probe (Fc), chronoamperometry, SEM, AFM, and HM detection for several modified electrodes prepared through CPE on GC electrodes from **L** solutions in 0.1 M TBAP/ACN in different conditions: **L** concentration ([**L**]), potential, and electric charge. The working electrodes were GC discs with either 3 mm diameter (GC3) or 6 mm diameter (GC6).

CME	[L] (mM)	Preparation Potential/ Electrode	Electric Charge (mC)	CME’s Characterization
1	1.3	1.3 V/GC3	0.5	Fc *^a^
2	1.3	1.3 V/GC3	1	Fc *^a^
3	1.3	1.6 V/GC3	0.5	Fc *^a^
4	1.3	1.6 V/GC3	1	Fc *^a^
5	1.3	1.6 V/GC3	1.3	Fc *^a^
6	1	1.3 V/GC3	1	Chronoamperometry, Fc *^b^
7	1	1.3 V/GC3	1.2	Fc *^a^
8	1	0.9 V/GC6	4	SEM, AFM, Raman
9	1	1.3 V/GC6	4	SEM, AFM, Raman
10	1	1.3 V/GC6	14	SEM, AFM, Raman

*^a^ Transfer of CME in Fc solution in 0.1 M TBAP/ACN; *^b^ HM detection.

**Table 2:** AFM topography images and line profile indentation acquired for CMEs obtained through CPE prepared at different potentials and charges: +0.9 V, 4 mC (CME 8), +1.3 V, 4 mC (CME 9), and +1.3 V, 14 mC (CME 10).

	AFM Topography	Line Profile Across Indentation	Thickness (nm)
**CME 8**	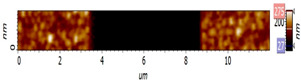	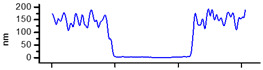	155
**CME 9**	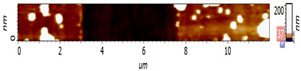	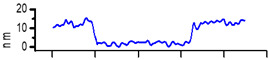	10
**CME 10**	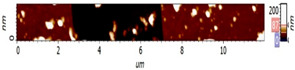	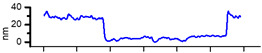	25

**Table 3:** Peak potentials (V) and electrochemical characteristics of CV and DPV peaks noticed on GC electrode in **L** solutions in 0.1 M TBAP/CH_3_CN during anodic and cathodic scans.

Peak	Method		Characteristics of the Peak
	DPV	CV	
a1	0.74	0.86	Irreversible
a2	1.04	1.14	Irreversible
a3	1.23	1.32	Irreversible
a4	2.27	2.55	Irreversible
c1	−1.22	−1.25	Reversible
c2	−1.77	−1.83	Reversible
c3	−2.16	−2.2	Reversible
c4	−2.94	−3.01	Irreversible

**Table 4:** Anodic (Epa) and cathodic (Epc) peak potentials of ferrocene in a 0.1 M TBAP/ACN solution, measured on bare electrodes and **L**-CMEs prepared through CPE under different preparation conditions in solution containing 1.3 mM of [**L**] in 0.1 M TBAP/ACN.

No.	Conditions\Fc Parameter	Epa (V)	10^−5^ · ipa (A)	Epc (V)	10^−6^ · ipc(A)	ΔEp *^1^	Ef *^2^
1	Bare electrode	0.138	3.025	0.037	−0.181	0.101	0.087
2	CPE at 1.3 V, 0.5 mC	0.183	1.887	0.015	−5.624	0.168	0.099
3	CPE at 1.3 V, 1 mC	0.205	1.356	−0.007	−1.882	0.212	0.099
4	CPE at 1.6 V, 0.5 mC	0.171	2.215	0.015	−7.34	0.156	0.094
5	CPE at 1.6 V, 1 mC	0.175	2.124	0.027	−7.138	0.148	0.104
6	CPE at 1.6 V, 1.3 mC	0.179	1.881	0.012	−5.619	0.167	0.094

*^1^ ΔEp = Epa − Epc; *^2^ Ef = (Epa + Epc)/2.

**Table 5:** Surface characteristics of L-CMEs prepared in different conditions through CPE on GC electrodes from **L** solutions with concentrations of 1 mM in 0.1 M TBAP/ACN.

L-CME	Condition	Morphology	Aggregation	Porosity
CME 8	+0.9 V, 4 mC	Fine, granular	Low	Low
CME 9	+1.3 V, 4 mC	Clustered, porous	Moderate	Moderate
CME 10	+1.3 V, 14 m	Dense, networked	High	High

## Data Availability

The data that support the conclusions of this paper are available in the manuscript and in the Supplementary Materials, but the raw data can be made available if requested (there are no confidential data for the manuscript and authors).

## Supplementary Materials

The following supporting information can be downloaded at https://www.mdpi.com/article/10.3390/molecules30193959/s1, Basic properties for 2-(azulen-1-yldiazenyl)-5-(thiophen-2-yl)-1,3,4-thiadiazole (**L**) and its characterization by appearance, melting point (m.p.), UV-Vis, ^1^H NMR, ^13^C-NMR, IR, MS, and elemental analysis; Figure S1: Reproducibility test: chronoamperograms for the individual experiments for CME preparation (used for metal detection) by CPE using a 1mC charge at a potential of +1.3 V; Figure S2: DPV curve (a) and RDE curves on GC recorded in solution of L in 0.1 M TBAP/ACN in [L] = 1 mM. Cathodic currents are presented as absolute values; Figure S3: CV (a) and DPV (b) curves at different concentrations of L in 0.1 M TBAP/ACN. All cathodic currents are presented as absolute values; Figure S4: Curves for **L** in TBAP/CH_3_CN 0.1 M at 1 mM concentration obtained through DPV (a) and CV (0.1 V/s) on different anodic and cathodic scan domains (b); all cathodic currents in the DPV are presented as absolute values. Table S1: Limiting currents* for each anodic (wa1 and wa4) and cathodic (wc1–wc4 in absolute values) RDE wave at different values of the electrode rotation rate ω (rpm); Table S2: Parameters of the linear dependences of the limiting currents (A) on the square root of the electrode rotation rate ω^1/2^ (rpm^1/2^) for anodic (wa1 and wa4) and cathodic* (wc1–wc4) RDE waves and linear regression parameters (intercept, slope, and Pearson correlation coefficient, R^2^); Table S3: Main characteristics of DPV stripping peaks (0.01 V/s) recorded in 0.1 M acetate buffer (pH 4.5) after 15 min of accumulation in solutions containing Cd(II), Pb(II), and Hg(II) for different concentrations ([HM]) in water; the modified electrodes were obtained through CPE at +1.3 V using an electrical charge of 1 mC in 1 mM **L** solution in 0.1 M TBAP/ACN.
