# Supplementary material for: Polymer Films of 2-(Azulen-1-yldiazenyl)-5-(thiophen-2-yl)-1,3,4-thiadiazole: Surface Characterization and Electrochemical Sensing of Heavy Metals
Source: Molecules. 2025 Oct 2;30(19):3959. doi: 10.3390/molecules30193959 (PMC12526234; doi:10.3390/molecules30193959)
Supplement: Supplementary file 1 [file molecules-30-03959-s001.zip › molecules-3842128-supplementary.pdf]

Supplementary Materials

Polymer Films of 2-(azulen-1-yldiazenyl)-5-(thiophen-2-yl)-1,3,4-thiadiazole: Surface Characterization and Electrochemical Sensing of Heavy Metals

Cornelia Musina (Borsaru) [1], Mihaela Cristea [2], Raluca Gavrilă [3], Oana Brîncoveanu [3], Florin Comănescu [3], Veronica Anăstăsoaie [3,*], Gabriela Stanciu [4], Eleonora-Mihaela Ungureanu [5,*]

Basic properties for 2-(azulen-1-yldiazenyl)-5-(thiophen-2-yl)-1,3,4-thiadiazole, (L) and its characterization by appearance, melting point (m.p.), UV-Vis, ^{1}H NMR, ^{13}C-NMR, IR, MS and elemental analysis:

Brown crystals, m. p. 212 ºC. UV-vis (MeOH), λmax (logε): 228 (4.39), 275 (4.18), 346 (3.96), 507 (4.51) nm. ^{1}H-NMR ($CDCl_3$, 500 MHz) δ 7.15 (dd, ^3J = 5.1, 3.7 Hz, 1 H, 4"-H), 7.48 (t,^3J = 4.7 Hz, 1 H, 3-H), 7.51 (d, ^3J = 5.1 Hz, 1 H, 5"-H), 7.54 (t, ^3J = 9.7 Hz, 1 H, 5-H), 7.64 (d, ^3J = 3.7 Hz, 1H, 3"-H), 7.67 (t, ^3J = 9.8 Hz, 1 H, 7-H), 7.89 (t, ^3J = 9.9 Hz, 1 H, 6-H), 8.38 (d, ^3J = 4.7 Hz, 1 H, 2-H), 8.39 (d, ^3J = 9.6 Hz,1 H, 4-H), 9.21 (d, ^3J = 9.6 Hz, 1 H, 8-H) ppm. ^{13}C-NMR ($CDCl_3$, 125 MHz) δ 123.0 (C-3), 127.1 (C-2), 128.1 (C-4"),129.5 (C-3"), 129.6 (C-1), 129.7 (C-2"), 129.9 (C-5), 130.0 (C-7), 133.6 (C-5"), 136.1 (C-8), 139.4 (C-4), 140.8 (C-6), 144.6 (C-8a), 146.9 (C-3a), 160.6 (C-5′), 181.0 (C-2′) ppm. IR (neat): 714, 742, 783, 839, 915, 1013, 1049, 1078, 1165, 1196, 1238, 1264, 1314, 1405, 1436, 1489, 1531, 1589, 1981, 2056, 2169, 2196, 2360, 2849, 2917, 2955, 3095 cm^{-1}. MS [ESI]: 323 [M+1].Calcd. for $C_{16}H_{10}N_4S_2$: C, 59.61; H, 3.13; N, 17.38. Found: C, 59.60; H, 3.15; N, 17.37.

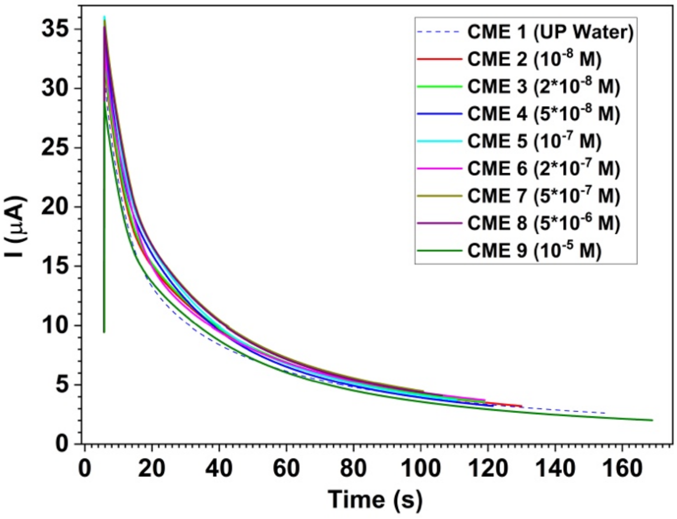

Figure S1. Reproducibility test: chronoamperograms for the individual experiments for CME preparation (used for metal detection) by CPE using a 1mC charge at a potential of 1.3 V.

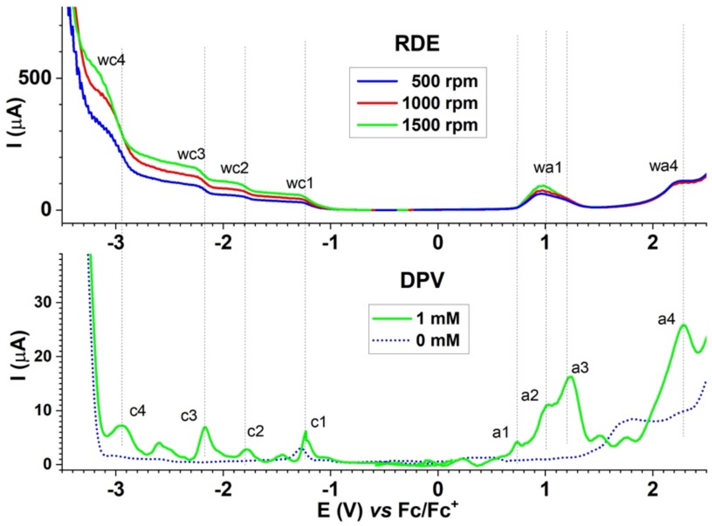

Figure S2. DPV curve (a) and RDE curves on GC recorded in solution of **L** in 0.1MTBAP/ACN; in [**L**] = 1 mM. Cathodic currents are presented as absolute values.

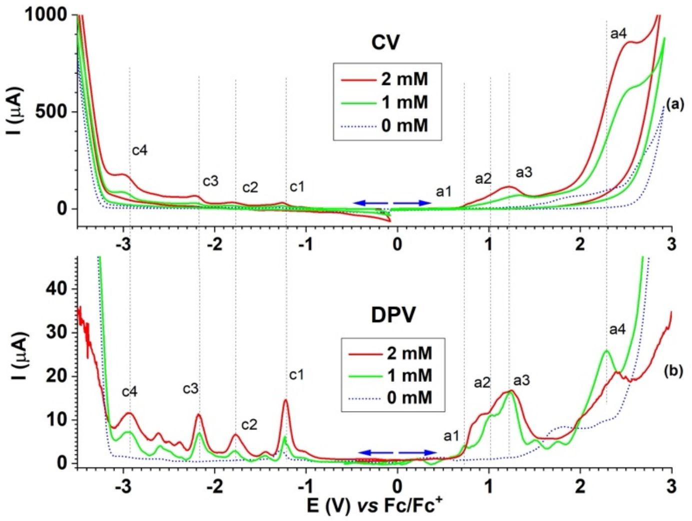

Figure S3. CV (a) and DPV (b) curves at different concentrations of **L** in 0.1 M TBAP/ACN. All cathodic currents are presented as absolute values.

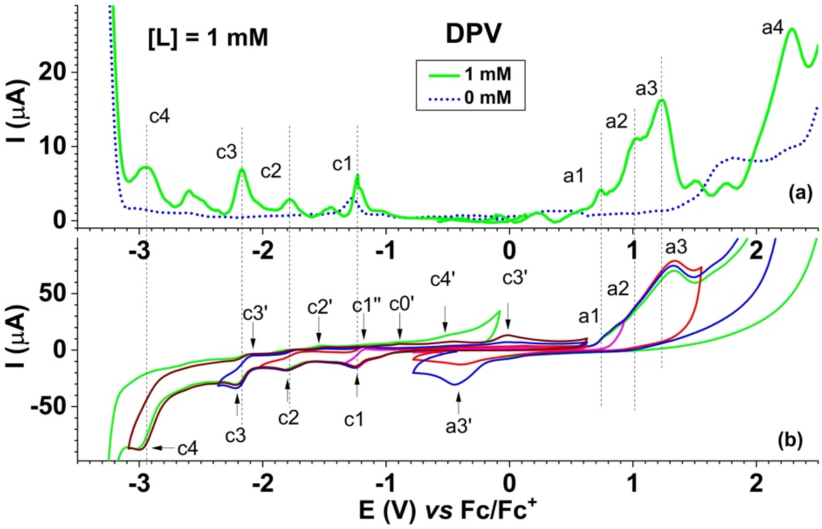

Figure S4. Curves for **L2548** in TBAP/CH3CN 0.1 M at 1 mM concentration obtained trough DPV (**a**) and CV (0.1 V/s) on different anodic and cathodic scan domains (**b**); all cathodic currents in the DPV are presented as absolute values.

Table S1. Limiting currents* for each anodic (wa1 and wa4) and cathodic (wc1-wc4 in absolute values) RDE waves at different values of the electrode rotation rate ω (rpm).

w (rpm)	ilwa1 (A)	ilwa4 (A)	ilwc1 (A)	ilwc2 (A)	ilwc3 (A)	ilwc4 (A)
500	6.29×10^{-5}	9.955×10^{-5}	3.302×10^{-5}	2.241×10^{-5}	8.149×10^{-5}	2.666×10^{-4}
1000	7.5×10^{-5}	9.275×10^{-5}	4.77×10^{-5}	3.201×10^{-5}	1.0839×10^{-4}	3.520×10^{-4}
1500	9.325×10^{-5}	9.725×10^{-5}	6.13×10^{-5}	4.57×10^{-5}	1.28×10^{-4}	4.143×10^{-4}

*Calculated as difference between the current at the starting point of the wave and its limiting current.

Table S2. Parameters of the linear dependences of the limiting currents (A) on the square root of the electrode rotation rate $\omega^{1/2}$ ($rpm^{1/2}$) for anodic (wa1 and wa4) and cathodic* (wc1-wc4) RDE waves and linear regression parameters (intercept, slope, and Pearson correlation coefficient, R^2).

Parameter	Intercept (A)	Slope (A*$rpm^{-1/2}$)	R^2
ilwa1 (A)	2.07×10^{-5}	1.80×10^{-6}	0.982
ilwa4 (A)	1.02×10^{-4}	-1.68×10^{-7}	-0.400
abs ilwc1 (A)	-5.66×10^{-6}	1.71×10^{-6}	0.998
abs ilwc2 (A)	-9.88×10^{-6}	1.39×10^{-6}	0.984
abs ilwc3 (A)	1.83×10^{-5}	2.83×10^{-6}	0.999
abs ilwc4 (A)	6.61×10^{-5}	8.99×10^{-6}	0.999

* In absolute value.

Table S3. Main characteristics of DPV stripping peaks (0.01 V/s) recorded in 0.1 M acetate buffer (pH 4.5) after 15 minutes of accumulation in solutions containing Cd(II), Pb(II) and Hg(II) for different concentration ([HM]) in water; the modified electrodes were obtained through CPE at +1.3 V using an electrical charge of 1 mC in 1 mM **L** solution in 0.1 M TBAP/ACN.

[HM]	Cd (II)		Pb (II)		Hg (II)	
(M)	i(A)peak	E(V)peak	i(A)peak	E(V)peak	i(A)peak	E(V)peak
10^{-8}	-	-	7.30×10^{-9}	-0.67	-	-
2×10^{-8}	-	-	1.54×10^{-7}	-0.67	-	-
5×10^{-8}	-	-	2.30×10^{-7}	-0.63	-	-
10^{-7}	-	-	2.13×10^{-7}	-0.60	-	-
2×10^{-7}	0	0	8.60×10^{-7}	-0.61/-0.55*	0.320×10^{-7}	0.22
5×10^{-6}	0.630×10^{-7}	-0.82	1.5710^{-6}	-0.60/-0.54*	0.194×10^{-6}	0.22
10^{-5}	0.117×10^{-6}	-0.81	2.49×10^{-6}	-0.59	0.438×10^{-6}	0.23

*The first value corresponds to Pb1 (-0.60 V) and the other (-0.54 V) correspond to amalgamated Pb (Pb2).
